# Recurrent tuberculosis and associated factors: A five - year countrywide study in Uzbekistan

**DOI:** 10.1371/journal.pone.0176473

**Published:** 2017-05-04

**Authors:** Jamshid Gadoev, Damin Asadov, Anthony D. Harries, Nargiza Parpieva, Katie Tayler-Smith, Petros Isaakidis, Engy Ali, Sven Gudmund Hinderaker, Gozalov Ogtay, Andrew Ramsay, Avazbek Jalolov, Masoud Dara

**Affiliations:** 1 World Health Organization country office in Uzbekistan, Tashkent, Uzbekistan; 2 State Post Graduate Institute of Medical Education, Tashkent, Uzbekistan; 3 International Union Against Tuberculosis and Lung Disease, Paris, France; 4 London School of Hygiene and Tropical Medicine, London, United Kingdom; 5 Republican Specialized Scientific-Practical Medical Center of Phthisiology and Pulmonology, Tashkent, Uzbekistan; 6 Medecins Sans Frontieres (MSF), Operational Center Brussels, Operational Research Unit, MSF-Luxembourg, Luxembourg, Luxembourg; 7 Centre for International Health, University of Bergen, Bergen, Norway; 8 WHO Regional Office for Europe, Joint Tuberculosis, HIV & Hepatitis Programme, Copenhagen, Denmark; 9 UNICEF, UNDP, World Bank and WHO Special Programme for Research and Training in Tropical Diseases (TDR), World Health Organization, Head quarter, Geneva, Switzerland; 10 University of St Andrews School of Medicine, Fife, Scotland, United Kingdom; 11 Ministry of Health of Republic of Uzbekistan, Tashkent, Uzbekistan; Universidad Nacional de la Plata, ARGENTINA

## Abstract

**Background:**

In Uzbekistan, despite stable and relatively high tuberculosis treatment success rates, relatively high rates of recurrent tuberculosis have recently been reported. Recurrent tuberculosis is when a patient who was treated for pulmonary tuberculosis and cured, later develops the disease again. This requires closer analysis to identify possible causes and recommend interventions to improve the situation. Using countrywide data, this study aimed to analyse trends in recurrent tuberculosis cases and describe their associations with socio-demographic and clinical factors.

**Method:**

Countrywide retrospective cohort study comparing recurrent tuberculosis patients with all new tuberculosis patients registered within the NTP between January 2006 and December 2010 using routinely collected data. Determinants studied were baseline characteristics and treatment outcomes.

**Results:**

Of 107,380 registered patients during the period January 2006 and December 2010, 9358 (8.7%) were recurrent cases. Between 2006 and 2008, the number of recurrent cases per annum increased from 1530 to 2081, then fell slightly thereafter from 2081 to 1888 cases. The proportion of all notified cases during this period increased from 6.5% to 9.9%. Factors associated with recurrent tuberculosis included age (35–55 years old), having smear positive pulmonary tuberculosis, residing in certain areas of Uzbekistan, having particular co-morbidities (including chronic obstructive pulmonary disease and HIV), and being unemployed, a pensioner or disabled. Recurrent tuberculosis patients also had a higher likelihood of having an unfavourable treatment outcome

**Conclusion:**

Despite signs of declining national tuberculosis notifications between 2006 and 2010, the relative proportion of recurrent cases appears to have increased. These findings, together with the identification of possible risk factors associated with recurrent tuberculosis, highlight various areas where Uzbekistan needs to focus its tuberculosis control efforts, particularly in light of the country’s rapidly emerging multi drug resistant tuberculosis epidemic.

## Introduction

Despite major advances in tuberculosis (TB) control, TB is still one of the largest public health challenges globally, and no less in Central Asia where TB incidence rates remain high [1]. The Republic of Uzbekistan, one of the 15 former Republics of the Soviet Union, is the most populous country in Central Asia and had an estimated TB incidence of 82 per 100,000 population in 2014 [1]. It is among the 18 high-priority countries for TB control in the WHO European region and one of the 27 high burden countries for multidrug-resistant TB (MDR-TB) globally [1].

Uzbekistan began a phased implementation of the WHO-recommended DOTS (directly observed treatment, short-course) strategy in 1998 and by 2005, DOTS had been rolled out countrywide, with case detection based mainly on passive case finding [2]. Over the last decade, Uzbekistan has made commendable strides in its TB control efforts: country data reported by the WHO indicate that the notification rate of new and recurrent TB cases declined almost 32% between 2006 and 2010 (from 91 to 62 per 100 000 population) [3], and treatment success rates have been stable and relatively high at around 83% (albeit just short of the 85% target set by the WHO). However, TB recurrence rates in the country are relatively high.

“Recurrent TB” patients are defined as patients who have previously been treated for TB, were declared cured or treatment completed at the end of their most recent course of treatment, and are now diagnosed with a recurrent episode of TB (either a true relapse due to reactivation of the disease or a new episode of TB caused by reinfection) [4]. Recurrent TB is an indicator of community control of TB and a proxy of TB drug-resistance. A study carried out during 2001–2002 in Republic of Karakalpakstan—an autonomous republic of Uzbekistan—showed that among 118 successfully treated TB patients, 36% were re-diagnosed with active TB within the next 22 months, with 52% of these re-diagnoses being sputum smear-positive for Acid Fast Bacilli (AFB). Patients classified as treatment completed had significantly higher smear-positive recurrence than those classified as cured [5].

The findings from studies conducted in other countries have shown that risk factors for recurrent TB include irregular drug intake, initial drug resistance, smoking, and alcoholism [6,7].

To date, there has been no analysis of country-wide data in Uzbekistan to describe the number and proportion of TB patients with recurrent disease or their trends over time. Moreover, risk factors associated with recurrent TB at the national level have not been described or analyzed in detail. The national TB control programme (NTP) uses a centralised individual-patient electronic database which allows for the detailed analysis of such data at a national level. Information on recurrent TB cases will be useful for the national TB control programme in Uzbekistan, and potentially other countries in the region, to consider interventions to address these challenges.

Using countrywide TB data from Uzbekistan, this study aimed to describe trends and risk factors for recurrent TB between 2006 and 2010. Specific objectives were to determine: i) the trend of recurrent TB annually over a five year period between 2006 and 2010 and ii) the association of socio-demographic, clinical characteristics and co-morbidities of patients with recurrent TB compared to those registered with new TB between 2006 and 2010.

## Methods

### Study design

This was a retrospective cohort study comparing recurrent TB patients with new TB patients registered within the NTP between January 2006 and December 2010. Determinants were baseline characteristics and treatment outcomes.

### Study setting

Part of the former Soviet Union until 1991, Uzbekistan is a country in Central Asia with a population of more than 31 million [8]. It is made up of twelve provinces (oblasts), one autonomous republic (the Republic of Karakalpakstan) and the capital city, Tashkent.

#### TB control activities

TB control activities are coordinated countrywide by the Republican Specialized Scientific-Practical Medical Center of Phthisiology and Pulmonology—essentially the National Tuberculosis Programme (NTP). All patients diagnosed with TB are treated free of charge within the NTP; there is no private sector for TB treatment. During the study period all registered TB patients received treatment in accordance with the WHO recommended DOTS strategy. At the province level, provincial TB hospitals provide TB control services under the supervision of the Ministry of Health (MoH), province State Health departments and the NTP.

After registration and initiation of treatment (category 1 treatment for new cases and category 2 treatment for recurrent TB cases), patients are hospitalized during the intensive phase of treatment (two months for new cases and three months for recurrent TB cases); thereafter the continuation phase of treatment (four months for new patients and five months for recurrent cases) is provided on an ambulatory basis [2]. Patients undergo sputum smear testing after treatment completion in the intensive phase and if the smears are negative for AFB patients are referred to primary health care facilities for the continuation phase of treatment. In certain situations (for example, a patient with smear negative pulmonary TB or a patient with childhood TB), the patients may receive full ambulatory treatment from the beginning of treatment. Treatment is prescribed by TB specialists during the intensive phase and by primary health care workers during the continuation phase. The duration of both phases of treatment may be extended based on sputum smear results.

At the time of the study, the country was covered by a network of TB laboratories that included two National Reference Laboratories (NRL) (one in Tashkent and one at the Republican TB dispensary in Nukus, Republic of Karakalpakstan), five bacteriological laboratories (where only AFB microscopy and culture using solid media are done) and more than 300 smear microscopy laboratories. None of these laboratories performed DNA fingerprinting of *Mycobacterium Tuberculosis* strains.

TB treatment is provided by Provincial TB hospitals/dispensaries at the province level and by TB dispensaries at both the district level (intensive phase) and the primary health care level (continuation phase) for drug-sensitive and multidrug-resistant TB (MDR-TB—resistant to both isoniazid and rifampicin). During the study, the Global Fund to Fight AIDS, Tuberculosis and Malaria (GFATM) was supplying all first-line anti -TB drugs countrywide and second-line drugs for pilot sites for the treatment of drug resistant tuberculosis (DR TB). Both drug-susceptible and drug-resistant TB patients are treated in accordance with the TB Order of the Ministry of Health of the Republic of Uzbekistan, which aligns with WHO-recommended guidelines [9].

#### NTP monitoring system

Since 2005, an Epi-Info based-TB-ESCM (Tuberculosis Electronic Surveillance and Case Management) system has been implemented countrywide for disease surveillance and case management. All diagnosed patients are individually recorded in this register and all of their clinical data are recorded here. Standardized treatment outcomes are monitored according to national and international recommendations.

### Study population

The study included all new and recurrent TB patients in Uzbekistan registered within the NTP and started on anti-TB treatment between January 2006 and December 2010.

#### Data sources, variables and data collection

The data source was the TB ESCM electronic register. Data variables included: TB registration number, socio-demographic characteristics at the time of TB diagnosis (age, sex, place of residence, occupation etc.), clinical characteristics at the start of TB treatment (TB type, category of TB—new and recurrent, and co-morbidities) and treatment outcomes. Data pertaining to this study were exported into EpiData (version 3.1, EpiData Association, Odense, Denmark).

#### Data analysis

Trends in recurrent TB were summarized using descriptive statistics. Baseline socio-demographic and clinical characteristics of recurrent TB patients were compared with new TB patients, and factors associated with recurrent TB determined by crude odds ratios (ORs) and adjusted ORs. Adjusted ORs were determined through multivariate logistic regression using a backward stepwise elimination approach until all remaining variables in the model were significant at *P* = 0.05 or less. All related *P*-values were based on the Walds test and 95% confidence intervals were used throughout. Data were analysed using EpiData Analysis software (version 2.2.2.182, EpiData Association, Odense, Denmark), Stata/SE (version 12; Stata Corporation, College Station, Texas 77845, USA) and Open Epi (Version 3.03a).

#### Ethics approval

Approval for this study was received from the National Ethics Committee under the Ministry of Health of Uzbekistan and from the Ethics Advisory Group of the International Union Against Tuberculosis and Lung Disease, Paris, France. The study satisfied the criteria for reports using routinely collected programmatic data set by the Médecins Sans Frontières Ethics Review Board (ERB), Geneva, Switzerland. During the analysis no patient identifying information was used. As this was a retrospective analysis of programme routine data, patient informed consent was not required.

## Results

### Trends in recurrent TB

Trends in recurrent TB cases are shown in Table 1. Between 2006 and 2008, the number of recurrent cases per annum increased from 1530 to 2081 (3.4% increase), falling slightly between 2008 and 2010 (2081 to 1888 cases) but remaining stable as a proportion of all notified cases during this period (9.4–9.9%).

**Table 1:** Trends in number of all notified tuberculosis cases and recurrent cases in Uzbekistan between 2006 and 2010.

Year	All notified cases of tuberculosis* N	Recurrent cases n (%)
**2006**	23534	1530 (6.5)
**2007**	22050	1904 (8.2)
**2008**	20990	2081 (9.9)
**2009**	20937	1955 (9.4)
**2010**	19869	1888 (9.6)
**Total**	107380	9358

* This includes all new cases and all retreatment cases (relapse, treatment after failure, return after loss to follow-up and others).

### Factors associated with recurrent TB

Based on the fact that our sample was extremely large (and there was therefore the need to balance statistical with clinical significance), an OR of 1.3 or more was used to single out factors most associated with recurrent TB. These factors included being an older adult (36–55 years old), having smear positive PTB, having particular co-morbidities (including chronic obstructive pulmonary disease (COPD) and HIV) and being unemployed, a pensioner or disabled Table 2. In the case of geographical residence, residing in the Navoi mining area, the Republic of Karakalpakstan, Bukhara province and the city of Tashkent was more strongly associated with recurrent TB than residing in other provinces (based on the magnitude of the respective ORs). Recurrent TB patients also had a higher likelihood of having an unfavourable treatment outcomes (died, treatment failure or loss to follow-up) compared with new TB cases.

**Table 2:** Association of socio-demographic and clinical characteristics of recurrent tuberculosis patients compared with all new tuberculosis (TB) patients in Uzbekistan, 2006–2010.

Variables	All new TB patients n (%)	Relapse patients n (%)	Crude OR (95%CI)	Adjusted OR^a^ (95%CI)	*P* value
**Total**	81016	9358	-	-	-
**Age (years)**					
Children (<15)	10875 (13)	212 (2)	0.2 (0.2–0.2)	0.8 (0.6–1.0)	0.05
Adolescent (15–18)	4095 (5)	252 (3)	0.5 (0.5–0.6)	0.9 (0.6–1.0)	0.07
Younger adults (19–35)	28884 (36)	3267 (35)	1	1	
Older adults (36–55)	22423 (28)	3810 (41)	1.5 (1.4–1.6)	1.3 (1.2–1.4)	<0.001
Elderly patients (>55)	14739 (18)	1817 (19)	1.1 (1.0–1.2)	1.0 (0.9–1.2)	0.59
**Sex**					
Male	47345 (58)	5669 (61)	1.1 (1.0–1.1)	1.2 (1.1–1.2)	<0.001
Female	33671 (42)	3689 (39)	1	1	
**Place of residence**					
Urban	25009 (31)	2939 (31)	1.2 (1.1–1.3)		
Rural	55977 (69)	5491 (59)	1		
Unknown	30 (<1)	928 (10)	-		
**Provinces**					
Republic of Karakalpakstan	9669 (12)	2198 (23)	3.2 (2.9–3.5)	2.0 (1.8–2.3)	<0.001
Tashkent city	5903 (7)	901 (10)	2.1 (1.9–2.4)	1.8 (1.6–2.1)	<0.001
Andijan province	7041 (9)	719 (8)	1.4 (1.2–1.6)	1.1 (1.0–1.3)	0.04
Bukhara province	3563 (4)	454 (5)	1.8 (1.5–2.0)	1.9 (1.7–2.2)	<0.001
Jizzakh province	3146 (4)	266 (3)	1.2 (1.0–1.4)	0.9 (0.8–1.1)	0.52
Kashkadarya province	7647 (9)	537 (6)	1	1	
Navoi province	2804 (3)	319 (3)	1.6 (1.4–1.8)	(0.9–1.2)	0.90
Namangan province	6995 (9)	709 (8)	1.4 (1.2–1.6)	1.4 (1.2–1.5)	<0.001
Samarkand province	8082 (10)	936 (10)	1.6 (1.4–1.8)	1.4 (1.3–1.6)	<0.001
Surkhandarya province	4203 (5)	154 (2)	0.5 (0.4–0.6)	0.5 (0.4–0.6)	<0.001
Syrdarya province	1949 (2)	214 (2)	1.5 (1.3–1.8)	1.5 (1.3–1.8)	<0.001
Tashkent province	7995 (10)	722 (8)	1.2 (1.1–1.4)	1.1 (1.0–1.2)	0.15
Fergana province	8056 (10)	758 (8)	1.3 (1.1–1.5)	1.2 (1.0–1.3)	0.001
Khorezm province	3771 (5)	438 (5)	1.6 (1.4–1.8)	1.2 (1.1–1.4)	0.003
Navoi mining company	184 (<1)	33 (<1)	2.5 (1.7–3.7)	4.9 (3.2–7.5)	<0.001
Unknown	8	0	-	-	-
**Co-morbidities**^b^					
None	65397 (81)	6404 (68)	1	1	
Diabetes mellitus	2568 (3)	289 (3)	1.1 (1.0–1.3)	0.5 (0.5–0.6)	<0.001
COPD	5398 (7)	826 (9)	1.6 (1.4–1.7)	1.2 (1.1–1.3)	<0.001
Hypertension	2837 (4)	347 (4)	1.2 (1.1–1.4)	0.9 (0.8–1.0)	0.02
Stomach-duodenal ulcer	720 (<1)	71 (<1)	1.0 (0.8–1.3)	0.6 (0.5–0.8)	<0.001
Psychological disorders	722 (<1)	57 (<1)	0.8 (0.6–1.1)	0.2 (0.1–0.3)	<0.001
HIV	710 (<1)	103 (1)	1.5 (1.2–1.8)	1.5 (1.2–1.9)	<0.001
Any oncological disease	146 (<1)	13 (<1)	0.9 (0.5–1.6)	0.4 (0.2–0.8)	0.008
Unknown	2518 (3)	1248 (13)	-	-	-
**TB type**					
PTB					
Smear positive	24480 (30)	4483 (48)	1.6 (1.-1.7)	1.8 (1.7–1.9)	<0.001
Smear negative	32240 (40)	3626 (39)	1	1	
No sputum/sputum result	1940 (2)	370 (4)	1.7 (1.5–1.9)	1.9 (1.6–2.2)	<0.001
EPTB	22356 (28)	879 (9)	0.3 (0.3–0.4)	0.6 (0.5–0.6)	<0.001
**History of contact with TB patient**					
No	75457 (93)	6630 (71)	1.1 (1.0–1.3)		
Yes	4810 (6)	361 (4)	1		
Unknown	749 (<1)	2367 (25)	-		
**History of imprisonment** ^d^					
No	36663 (45)	3128 (33)	1		
Yes	368 (<1)	57 (<1)	1.8 (1.4–2.4)		
Unknown	43985 (54)	6173 (66)	-		
**Occupational status**					
Worker	10908 (13)	568 (6)	1	1	
Pupil/student	10661 (13)	208 (2)	0.4 (0.3–0.4)	0.8 (0.6–1.0)	0.04
Handicapped	3052 (4)	1274 (14)	8.0 (7.2–8.9)	10.0 (8.9–11.2)	<0.001
Pre-school age	2938 (4)	21 (<1)	0.1 (0.1–0.2)	0.3 (0.2–0.5)	<0.001
Pensioner	11602 (14)	1081 (12)	1.8 (1.6–2.0)	2.1 (1.8–2.4)	<0.001
Jobless	39283 (48)	4200 (45)	2.1 (1.9–2.2)	2.1 (1.9–2.3)	<0.001
Unknown	2572 (3)	2006 (21)	-	-	-
**Treatment outcomes**					
Treatment success	70445 (87)	6810 (73)	1		
Died	3493 (4)	936 (10)	2.8 (2.6–3.0)		<0.001
Failure	1858 (2)	480 (5)	2.7 (2.4–3.0)		<0.001
Loss to follow up	4162 (5)	841 (9)	2.1 (1.9–2.3)		<0.001
Transferred out	1058 (1)	291 (3)	2.8 (2.5–3.2)		<0.001

COPD, Chronic Obstructive Pulmonary Disease; PTB, Pulmonary TB; EPTB, Extrapulmonary TB; OR, Odds Ratio, CI, Confidence Interval

^a^ Adjusted odds ratios only presented for variables included in the multivariate model; 84389 records included in the multivariate model due to missing records for some variables

^b^ Co-morbidities are generally self-reported by patients, and only one co-morbidity per patient can be reported in the electronic reporting system

^d^ History of imprisonment not included in the multivariate model due to large amounts of unknown data

Due to concerns around the completeness of our data on co-morbidities, our multivariate analysis exploring risk factors associated with recurrent TB compared with new TB, was also run excluding the co-morbidity variable. This did not have any bearing on the specific factors found to be associated with recurrent TB nor did it significantly affect the strength of these associations.

## Discussion

This is the first countrywide study from Uzbekistan to report on annual trends in recurrent TB and associated risk factors. Despite a promising year-on-year decline in national TB notifications between 2006 and 2010, there has been an overall slight increase in the relative proportion of recurrent TB for the same period from 6.5% to 9.9%. These findings, together with the identification of possible risk factors associated with recurrent TB, raise a number of important issues and highlight various areas where Uzbekistan needs to focus its TB control efforts.

Recent research carried out in Republic of Karakalpakstan found that, although three-quarters of new cases were “successfully” treated, a third of these “successes” were later re-diagnosed with TB. Recurrence of TB was particularly common among patients whose initial disease was multidrug resistant. Previous TB treatment was also associated with an increased risk of disease recurrence [5].

While our study has indicated that the proportion of recurrent cases in Uzbekistan plateaued at around 9% between 2008 and 2010, recent WHO country estimates suggest that this proportion has nearly doubled since then, being at around 17% in 2014 [1]. Restricted access to programmatic data beyond 2010 precluded us from being able to examine more recent trends in relapse TB. Not only is recurrent TB associated with worse treatment outcomes compared with new TB [5, 10]—as noted in our study—but one of the major public health concerns is the associated risk of drug-resistant TB, particularly MDR-TB. In the 2010 global surveillance for drug-resistant TB, it was estimated that 7.9% of recurrent cases in the world had MDR-TB [11]. In a country like Uzbekistan where rates of MDR-TB are among the highest in the world (23% among incident cases and 62% among retreatment cases [12]), tackling what appears to be rising rates of recurrent TB, is thus of utmost importance.

Our study indicated a number of factors that seem to be associated with recurrent TB, although in view of the substantial size of the study and the need to balance clinical and statistical significance, we have been selective in teasing out the factors that we deem to be important clinically and from a public health perspective. First, the risk of recurrent TB was highest among older adults (36–55 years). Unaware of any previous studies that have identified particular age groups as being more at risk of recurrent TB, we postulate that this finding is likely linked to other co-determinants such as poor treatment adherence, smoking [13], alcohol intake and co-existing morbidities—factors that we were unable to assess and control for in this study. Further investigation would be needed to establish these reasons so that more targeted measures aimed at mitigating these factors could be implemented.

Second, the risk of recurrent TB was higher in certain parts of the country, in particular in the Navoi mining company. This particular finding may be related to an increased incidence of silicosis among miners (linked to the presence of silica dust in the mines), and silicosis has been shown to be a risk factor for TB [14].

In other parts of the country we can only speculate, but the geographic disparities may reflect several issues, like: i) variations in drug resistance prevalence and resistance patterns (the highest rates of MDR-TB have been reported in the Republic of Karakalpakstan for example [12]), ii) differences in the performance of local TB control activities and local primary health care services, iii) the availability of first and second line anti TB drugs in the open market, and/or iv) differences in patient characteristics (e.g. socio-economic status, tobacco use, alcohol consumption, population movement and migration) [15, 16]. Operational research at the level of the province, including qualitative research methods, may help to identify these specific factors.

Third, certain co-morbidities were found to be associated with an increased risk of recurrent TB, including HIV and COPD. HIV is well known to increase the risk of recurrent TB both in high and low TB burden settings, [7, 17–19], although reinfection rather than true relapse is reported to be more common among HIV-infected individuals than non-infected persons [20]. Uzbekistan has a low HIV prevalence (0.2%) and therefore HIV is unlikely to be a major contributor of recurrent TB overall [21, 22]. However, the HIV epidemic in Uzbekistan is far from abating and high risk groups for HIV (in particular injecting drug users, sex workers and prisoners) [23] might be ‘hot spots’ for recurrent TB. COPD was also found to be associated with recurrent TB, corroborating the findings from other studies [24, 25]. COPD may increase the risk of recurrent TB because of fibrotic changes in the lung and reduced anti-TB drug penetration into the lung tissue [26]; evidence also suggests that high dose oral corticosteroids and oral β-agonist medications used for COPD may weaken the essential defense mechanism against *M*. *tuberculosis* in the airway and lung parenchyma [27]. Further study is nonetheless needed to better understand the link between COPD and recurrent TB.

Surprisingly, diabetes mellitus (DM) was not found to be associated with an increased risk of recurrent TB, in fact the converse was found: DM was found to be associated with a lower risk of recurrent TB. This is counter to substantial evidence showing that DM increases the risk of TB and also recurrent TB [28]. We suspect that this is a reflection of the incompleteness of our data on DM, primarily linked to two factors: i) first the electronic reporting system used in Uzbekistan only allows one co-morbidity per patient to be reported, despite the fact that patients may of course have more than one important co-morbidity. As such, some patients with known DM may not be reported as having this condition; ii) co-morbidities are generally self-reported by patients and given that half of the DM cases worldwide do not know that they have DM, it may go unidentified and thus unreported for TB patients.

Fourth, being disabled, a pensioner or unemployed was associated with an increased risk of recurrent TB, particularly being disabled. Once again, we can only speculate but we suspect that this is probably linked to patient-related characteristics (e.g. pre-existing co-morbidities, poor access to anti-TB drugs, poor treatment adherence) [29].

Finally, having smear positive pulmonary TB was associated with a high risk of recurrent TB compared with having smear negative pulmonary TB or extrapulmonary TB, mirroring the findings from other studies [5] and showing a similar situation to that seen in other central Asian countries [1]. Smear-positive TB at baseline was associated with recurrent TB—unfortunately we do not have robust data for the question about smear-positive TB after the intensive phase of treatment and therefore cannot comment here on the association with recurrent TB

One question that our data was not able to answer was whether recurrent TB cases were relapse TB or caused by exogenous reinfection. Evidence suggests that in countries with a low TB burden, TB recurrence is usually caused by relapse [30], whereas in countries with a high TB burden the principle cause is reinfection [31], especially when high levels of HIV infection co-exist [20]. In countries with a medium TB burden (like Uzbekistan), relapse and reinfection are both thought to play a role [32], with their relative contribution depending on the prevalence of epidemiological risk factors and the microbiological features of circulating *M*. *tuberculosis* strains. Relapse TB tends to reflect inadequacies in clinical management (linked to drug resistance, poor treatment adherence, poor drug quality, or inadequate treatment regimens for instance); while reinfection is often a result of a high prevalence of infectious TB in the community, low levels of immunity among individuals having completed TB treatment, HIV infection, or specific characteristics of circulating M. *tuberculosis* strains [5,12]. Further investigation would be needed to ascertain the relative contribution of relapse TB and reinfection to recurrent TB in Uzbekistan, together with the specific reasons. At any rate, given what appears to be a parallel rise in recurrent TB and MDR-TB in the country, we strongly suspect that MDR-TB has a large part to play in recurrent TB (true relapse and reinfection).

The main strength of this study was that it was a countrywide study and therefore nationally representative. Performing a risk factor analysis at the national level was also made possible by the fact that Uzbekistan has implemented a TB surveillance system that captures individual patient-level data, rather than aggregate data as in many other national TB programmes.

There were several study limitations. First, the study relied on routinely collected data which may have been subject to incompleteness or inaccurate capture. Indeed, substantial amounts of data were incomplete for certain variables such as ‘history of imprisonment’, restricting our ability to assess the association of such factors with recurrent TB. Second, this evaluation was limited to the available data in Uzbekistan’s national TB surveillance system and as such data on factors such as smoking, alcohol and injecting drug use were not routinely recorded. Third, the study design that we used did not involve the follow-up of ‘successfully treated’ patients to ascertain who and who did not develop recurrent disease; in Uzbekistan TB patients presenting for repeat episodes of treatment are not given the same unique identifier each time and therefore their treatment history cannot be accurately tracked in the electronic register. Instead, we compared new TB cases with recurrent cases in terms of associated factors, the limitation with this design being that a proportion of the new cases will have subsequently developed recurrent TB (i.e. their risk profile might be no different from a registered recurrent case). In this way, our study likely underestimated the strength of association of identified risk factors with recurrent TB. It also precluded any assessment of how long after “successful treatment”, patients develop recurrent TB. Finally, restricted access to data unfortunately precluded us from being able to examine national trends in recurrent rates of TB in Uzbekistan up until the current time. Reference to recent WHO estimates was thus our only means of gauging how the situation seems to have progressed since 2010.

The findings of our study have a number of implications. First, given the association between HIV and recurrent TB, ensuring that all TB patients are routinely screened for HIV as per the national guidelines in Uzbekistan is important. In addition, HIV co-infected TB patients not already on antiretroviral treatment (ART) should be started on ART as soon as possible after starting anti-TB treatment, together with co-trimoxazole preventive therapy. Both interventions are known to reduce recurrent TB [33–35]. Second, among COPD patients that have a history of previous TB, regular monitoring for the development of pulmonary TB seems prudent, especially among those receiving high doses of oral corticosteroids. Third, data capture on co-morbidities needs to be revised in the national TB electronic register so that multiple co-morbidities per patient can be simultaneously captured. Such data are important for understanding the relationship between different co-morbidities and TB, and for devising more effective responses to deal with inter-related burdens of diseases. Finally, given what seem to be rising rates of recurrent TB in Uzbekistan, reliance on end-of-treatment outcomes to judge the potential effectiveness of DOTS in controlling TB may no longer be appropriate. Instead, implementing and expanding the case management of drug resistant TB might be a plausible consideration [36].

In conclusion, this study shows that despite signs of declining national TB notifications between 2006 and 2010, the relative proportion of recurrent TB cases appears to be climbing. These findings, together with the identification of possible risk factors associated with recurrent TB, highlight various areas where Uzbekistan needs to focus its TB control efforts, particularly in light of the country’s rapidly emerging MDR-TB epidemic.

## Supporting information

S1 File UTO_2006_10_Sputum_selected.rec (REC)
